# Antibacterial Activity and Multi-Targeting Mechanism of Dehydrocorydaline From *Corydalis turtschaninovii* Bess. Against *Listeria monocytogenes*

**DOI:** 10.3389/fmicb.2021.799094

**Published:** 2022-01-11

**Authors:** Gowoon Kim, Yijuan Xu, Jiarong Zhang, Zhongquan Sui, Harold Corke

**Affiliations:** ^1^Department of Food Science and Technology, Shanghai Jiao Tong University, Shanghai, China; ^2^Biotechnology and Food Engineering Program, Guangdong Technion – Israel Institute of Technology, Shantou, China; ^3^Faculty of Biotechnology and Food Engineering, Technion-Israel Institute of Technology, Haifa, Israel

**Keywords:** foodborne pathogen, phytochemicals, alkaloids, antibacterial mechanism, proteomic analysis

## Abstract

*Listeria monocytogenes* is a foodborne pathogen, with relatively low incidence but high case-fatality. Phytochemicals have been recognized as a promising antimicrobial agent as an alternative to synthetic chemicals due to their safety and high efficacy with multi-target sites. This study identified and characterized a novel antibacterial agent, dehydrocorydaline, in the *Corydalis turschaninovii* rhizome using HPLC-LTQ-Orbitrap-HRMS, and its antibacterial effect with lowest MIC (1 mg/mL) and MBC (2 mg/mL) values. In addition, an *in vitro* growth kinetic assay, cytoplasmic nucleic acid and protein leakage assay, and observation of morphological changes in bacterial cells supported the strong antibacterial activity. Dehydrocorydaline also displayed effective inhibitory effects on biofilm formation and bacterial motility. In order to investigate the potential antibacterial mechanism of action of dehydrocorydaline against *L. monocytogenes*, label-free quantitative proteomics was used, demonstrating that dehydrocorydaline has multiple targets for combating *L*. *monocytogenes* including dysregulation of carbohydrate metabolism, suppression of cell wall synthesis, and inhibition of bacterial motility. Overall, this study demonstrated that dehydrocorydaline has potential as a natural and effective antibacterial agent with multi-target sites in pathogenic bacteria, and provides the basis for development of a new class of antibacterial agent.

## Introduction

*Listeria monocytogenes* is the causative agent of listeriosis, which is of relatively low incidence but presents a high case-fatality rate (20–30%) and causes serious disease such as gastroenteritis, meningitis, sepsis, and encephalitis (Radoshevich and Cossart, 2018). It is lethal to pregnant women, fetuses, neonates, elderly people, and immunocompromised people (Radoshevich and Cossart, 2018). In particular, due to depressed cellular immunity in maternity and placental tropism of *L. monocytogenes*, pregnant women and fetuses are at greater risk of listeriosis that may lead to miscarriage, fetal death, septicemia, meningoencephalitis, and neonatal death (Abram et al., 2003; Madjunkov et al., 2017). In China, 211 cases of listeriosis were reported from 2013 to 2017 with mortality rates of 16% for non-perinatal and 31% for perinatal patients (Li et al., 2019). The outbreak of listeriosis is mainly associated with consumption of contaminated food products including dairy products, meat products, and some ready-to-eat (RTE) foods. Low levels of *L. monocytogenes* contamination in food (10^4^–10^6^ bacteria/g of food intake) can lead to clinical symptoms (Madjunkov et al., 2017). Unlike most other foodborne pathogens, *L. monocytogenes* is able to survive in hostile environments such as low moisture, high salinity (10% NaCl), acidity (pH 4.5), and cold (Jordan and McAuliffe, 2018). In addition, it possesses inherent resistance to some antibiotics (cefotaxime, cefepime, fosfomycin, oxacillin, and lincosamide) and has developed multiple mechanisms of resistance to antibacterial agents by mutation, horizontal antibiotic gene transfer, and biofilm formation (Lungu et al., 2011; Olaimat et al., 2018). *Listeria monocytogenes* biofilm, which is a microbial aggregate embedded in a self-produced matrix containing extracellular polymeric substances (proteins, nucleic acids, and polysaccharides), is highly resistant to antibiotics compared to planktonic cells (Oloketuyi and Khan, 2017). This resistance is attributed to poor penetration, inactivation of antimicrobials in the biofilm matrix, and a change of bacterial metabolic state to dormancy (de la Fuente-Núñez et al., 2012). These abilities of *L. monocytogenes* are considered to be a major issue in regard to its significance in public health and food safety. Therefore, developing effective methods for the control of resistant strains and biofilms is crucial.

In the last decade, plant-derived compounds have been widely investigated as antimicrobial agents (Papuc et al., 2017). Many phytochemical compounds are produced in response to biotic stress from pests and diseases, and possess diverse chemical structures, contributing to their potential high efficacy against multi-target sites in pathogenic bacteria (Ma et al., 2019). Furthermore, compared to most synthetic antimicrobials, they are relatively safer and more acceptable to consumers (Silva et al., 2016; Shin et al., 2018). These features support phytochemicals as promising antimicrobial agents as alternatives to synthetic chemicals including antibiotics.

*Corydalis turtschaninovii* Bess. belongs to the Papaveraceae family, and is widely distributed in East Asia including China, South Korea, and Japan. The *C. turtschaninovii* rhizome contains abundant alkaloids with biologically important activities such as analgesic (Zhang et al., 2014), anti-amnesic (Hung et al., 2008), anti-inflammatory (An et al., 2009), and anti-cancer effects (Kim et al., 2012; Lee et al., 2017). However, the antibacterial properties of *C. turtschaninovii* and its antibacterial compounds remain largely unknown. Our preliminary study demonstrated that the rhizome of *C. turtschaninovii* exhibited potent antibacterial properties (Kim et al., 2020), showing its potential to identify and develop a new antibacterial agent that will be expected to have multiple target sites in bacterial cells, cost efficiency, and low toxicity.

In the present study, we aimed to identify major compounds in crude extract of the *C. turtschaninovii* rhizome using liquid chromatography coupled with hybrid linear ion trap quadrupole-orbitrap mass spectrometry (HPLC-LTQ-Orbitrap-MS/MS) and to evaluate antibacterial activity of identified compounds against *L. monocytogenes*. To the best of our knowledge, this study is the first to report antibacterial activity of dehydrocorydaline found in *C. turtschaninovii*. Moreover, we also investigated the possible mechanism of antibacterial action of dehydrocorydaline on *L. monocytogenes* using label-free quantitative proteomic analysis with confirmation by qRT-PCR. By combining the qualitative analysis of phytochemical compounds in *C. turtschaninovii* and proteomics results, this study suggested dehydrocorydaline as a novel antibacterial compound and provided detailed multitargeted molecular mechanisms of antibacterial action of dehydrocorydaline against *L. monocytogenes.*

## Materials and Methods

### Chemical Reagents, Bacterial Strains, and Culture Conditions

Columbamine, corydaline, coptisine, corypalmine, dehydrocorydaline, jattorrhizine, oxoglaucine, palmatine, protopine tetrahydropalmatine, and yuanhunine were purchased from Chengdu Refmedic Biotech Co., Ltd. (Chengdu, China). Berberine was bought from Aladdin Reagents Co., Ltd (Shanghai, China). HPLC-grade methanol and formic acid were purchased from Sigma-Aldrich (Shanghai, China). *Listeria monocytogenes* ATCC 7644, *Staphylococcus aureus* ATCC 25923, *Escherichia coli* ATCC 25922, and *Salmonella enterica* Enteritidis ATCC 13076 were purchased from American Type Culture Collection (ATCC) and stored in our laboratory with 20% glycerol at −80°C. The strain was cultured in tryptic soy broth (TSB; Oxoid, Basingstoke, United Kingdom) medium at 37°C and 180 rpm.

### Preparation of Plant Extract and Identification of Phytochemical Compounds

The rhizome of *C. turtschaninovii* was obtained from Lishui, Zhejiang province, China (Specimen no. CT180320 in Shanghai Jiao Tong University). The ground rhizome (100 g) was extracted according to the procedure of Kim et al. (2020), yielding 4 g of dried extract. Major phytochemical compounds in the extract were identified using HPLC-LTQ-Orbitrap-MS/MS analysis, shown in the Supplementary Figures 1–3 and Supplementary Table 1.

### Determination of Minimum Inhibitory Concentration and Minimum Bactericidal Concentration

Minimum Inhibitory Concentration (MIC) and Minimum Bactericidal Concentration (MBC) were determined using a broth microdilution method (Kim et al., 2020). Each bacterial strain (1 ×10^6^ CFU/mL) was incubated with identified compounds at concentrations ranging from 0.01 to 2 mg/mL in a 96-well microplate for 24 h at 37°C. The concentration of the compound with no visible bacteria growth was regarded as the MIC. The wells with no visible bacterial growth were taken for colony counting and MBC was determined as the lowest concentration with no colony growth on a Mueller-Hinton (MH; Oxoid, Basingstoke, United Kingdom) agar plate.

### Growth Inhibition Assay

Bacteria (1 ×10^6^ CFU/mL) at the exponential phase were cultured in MH broth supplemented with various concentrations of dehydrocorydaline (0, 0.1, 0.25, 0.5, 1, 2 and 4 mg/mL) at 37°C for 24 h. At the selected time interval, the OD_600_ of cell suspension was determined by spectrophotometer (Molecular Devices, San Jose, CA, United States).

### Nucleic Acid and Protein Leakage Assay

Cytoplasmic nucleic acid and protein released from bacterial cells treated with dehydrocorydaline were determined to evaluate the integrity of the cell membrane (Chen et al., 2020). *Listeria monocytogenes* cells at the exponential growth phase were harvested by centrifugation at 2500 × *g* for 10 min, washed with phosphate buffered saline (PBS) three times, resuspended in PBS (1 ×10^6^ CFU/mL) containing different concentrations of dehydrocorydaline (0, MIC, and MBC), and incubated at 37°C for 4 h. The cell suspension was collected at different time intervals (0, 1, 2, and 4 h) and filtered through a 0.22 μm Millipore filter. The nucleic acid released from bacterial cells was measured by UV visible spectrophotometer (Allsheng, Hangzhou, China) with optical density at 260 nm. The amount of protein leakage was quantified by BCA protein assay (Takara Bio, Mountain View, CA, United States).

### Transmission Electron Microscopy

*Listeria monocytogenes* cells at exponential growth phase were treated with dehydrocorydaline (0, 1 and 2 mg/mL) for 4 h at 37°C. The harvested cells were washed with PBS three times and then resuspended in 2.5% glutaraldehyde (v/v in PBS) for 12 h at 4°C. Subsequently, the cells were gradually dehydrated in a series of graded ethanol concentrations (30, 50, 70, 80, 90, 95, and 100%) for 10 min. The sample was subsequently coated with formvar film, air-dried, and stained with phosphotungstic acid. The film was loaded using transmission electron microscopy FEI T12 equipped with an AMT XR51 CCD camera system (FEI, Hillsboro, OR, United States).

### Reactive Oxygen Species Measurement

Intercellular Reactive Oxygen Species (ROS) level in *L. monocytogenes* was determined using a 2′,7′-dichlorodihydrofluorescein diacetate assay (DCFH-DA, beyotime, Shanghai, China) (Li et al., 2016). Bacteria from overnight culture were collected by centrifugation at 2500 × *g* for 10 min and washed with PBS three times. The cells were resuspended in PBS (OD_600_ = 0.3) and dehydrocorydaline was added at final concentrations of 0, 0.25, 0.5, 1, and 2 mg/mL. After the incubation for 1 h, the bacteria were collected by centrifugation and washed with PBS three times. Then, the bacteria were resuspended in PBS and placed into each well of a 96-well microplate and 10 μM DCFH-DA was also added to each well. The plate was incubated in the dark for 30 min and at 37°C. Fluorescence was detected using a microplate reader (Molecular Devices) at an excitation wavelength of 488 nm and an emission wavelength of 525 nm.

### Antibiofilm Formation

Inhibition of biofilm formation was determined by using a crystal violet assay (Li et al., 2018). Briefly, *L. monocytogenes* (1 ×10^7^ CFU/mL) were inoculated into wells in a 96-well microtiter plate (Corning, Corning, NY, United States) containing different concentrations (0, 0.1, 0.25, 0.5, and 1 mg/mL) of dehydrocorydaline. After incubation at 37°C for 24 h, the planktonic cells were removed, and the wells were washed three times with sterile water. Subsequently, 200 μL of 0.1% (w/v) crystal violet (Aladdin, Shanghai, China) was added to each well, stained for 10 min, and removed. The stained biofilm was dissolved in 200 μL of 33% (v/v) glacial acetic acid (Sigma-Aldrich, Darmstadt, Germany), and the biofilm was quantified by measuring the absorbance at 595 nm using a microtiter reader (Molecular Devices, San Jose, CA, United States).

### Biofilm Metabolic Activity

The metabolic activity of biofilm was determined by a 3-[4,5-dimethylthiazol-2-yl]-2,5 diphenyl tetrazolium bromide (MTT) assay (Goswami et al., 2014). The preparation of bacterial biofilm is referred to in section “Reactive Oxygen Species Measurement.” The biofilm was treated with 100 μL of MTT solution (5 mg/mL) and incubated in the dark for 3 h and at 37°C. After removal of the MTT solution, 100 μL of DMSO was added into each well and the absorbance at 570 nm was measured.

### Anti-motility

Swimming and swarming motility assays were performed according to Li et al. (2018). A total of 3 μL of overnight culture of *L. monocytogenes* was placed in the center of the swimming (10 g/L tryptone, 5 g/L NaCl, and 0.3 % agar) and swarming (25 g/L LB, 0.5 g/L glucose, and 0.5% agar) plates containing various concentrations (0, 0.1, 0.25, 0.5, and 1 mg/mL) of dehydrocorydaline. After incubation at 37°C for 24 h, the motility was observed.

### Label-Free Quantitative Proteomics Analysis

#### Dehydrocorydaline Treatment of *Listeria monocytogenes*

*Listeria monocytogenes* cells (1 ×10^6^ CFU/mL) at the exponential growth phase were treated with dehydrocorydaline at sub-MIC (0.25 mg/mL) for 2 h and at 37°C, and cells without treatment were regarded as a control (each group in three biological replicates). The untreated or treated bacteria were harvested using centrifugation (800 × *g*, 10 min, 4°C) and were washed with PBS three times.

#### Protein Extraction, Quantification, Digestion, and Peptide Desalination

The proteins of untreated or treated cells were extracted using a Tris-Phenol protein extraction assay (Shi et al., 2018). The proteins were quantified by a bicinchoninic acid (BCA) protein assay kit (Beyotime, Shanghai, China). The protein digestion was conducted according to the procedure of Shi et al. (2018), with minor modification. Briefly, 200 μg of protein was treated with Tris(2-carboxyethyl)phosphine (TCEP) at a final concentration of 10 mM and incubated at 37°C for 1 h. After adding iodacetamide (IAA) at a final concentration of 40 mM and incubating for 40 min in the dark, the sample was treated with six volumes of cold acetone at −20°C until precipitate forms. After the removal of acetone, precipitated protein was resuspended with 10 mM triethylammonium bicarbonate (TEAB) buffer, digested with trypsin (Promega, Madison, WI) in a 1:50 ratio (protein: trypsin) at 37°C overnight. The peptides were dried, resuspended with 2% acetonitrile and 0.1% trifluoroacetic acid, desalted using Sep-Pak cartridges (Waters, Milford, MA, United States), and vacuum dried.

#### LC-MS/MS Analysis

An Easy-nLC system coupled with Q Exactive mass spectrometer (Thermo Fisher, Fairlawn, NJ, United States) through a nanoES ion source was used for analysis. Peptide (5 μg) was loaded onto a C_18_-reversed phase column (75 μm ×100 mm, 3 μm, Thermo Scientific, San Jose, CA, United States) in buffer A (2% acetonitrile and 0.1% formic acid) and separated with a linear gradient of buffer B (80% acetonitrile and 0.1% formic acid) at a flow rate of 250 μL/min controlled by IntelliFlow technology. An electrospray voltage of 1.8 kV was used, and the temperature was set to 275°C. The MS and MS/MS of peptide segments were acquired, dynamically selecting the top 20 most abundant precursor ions from a survey of full-scan MS spectra (*m/z* 350-1300) with full-scan MS resolution at 70,000, followed by higher-energy collisional dissociation (HCD) fragmentation (MS/MS) scans with resolution at 17,500. One microscan was recorded using dynamic exclusion duration of 18 s. Normalized collision energy was 30 eV.

#### Data Analysis

MS/MS spectra were searched against *L. monocytogenes* EGD-e using the Uniprot database and decoy database Mascot 2.2 (Matrix Science, London, United Kingdom) embedded in Proteome Discoverer 1.4 (Thermo Electron, San Jose, CA, United States). The search parameters were set as tryptic digestion with two missed cleavages, 0.1 Da of MS/MS tolerance, carbamidomethylation at cysteine residues as a fixed modification, and oxidation at methionine residues as variable modifications. The minimum peptide length was set to 6, and peptide spectral matches were validated based on false discovery rate (FDR) at 1%. The label-free quantification was performed based on the peak intensity of each sample (control and treatment group in three biological replicates) and the statistical significance was calculated using the Student’s *t*-test. Proteins with the thresholds of fold change (≥1.2 or ≤0.83) and *P* < 0.05 were considered to be differentially expressed. Gene Ontology (GO^1^) and the Kyoto Encyclopedia of Genes and Genomes database (KEGG^2^) were used to investigate the molecular function and metabolic pathway of all differentially expressed proteins.

### RNA Extraction and Quantitative Real-Time PCR

Total RNA was extracted from untreated or dehydrocorydaline-treated cells (0.75 mg/mL) using a total RNA extraction kit (Takara Bio, Beijing, China) and converted into cDNA using a cDNA synthesis kit (Takara Bio). Quantitative real-time PCR analysis was performed on a CFX96 Real-Time PCR System (Bio-Rad, Hercules, CA, United States) using a SYBR Green qPCR Master Mix (Takara Bio), primers (Supplementary Table 2), and 1 ng of cDNA template. The following conditions were used: initial denaturation at 95°C for 30 s, 40 cycles of 95°C for 5 s, and 60°C for 30 s, followed by a melting curve at 95°C for 15 s, 60°C for 60 s, 30°C for 95 s, and finally at 95°C for 15 s. Data were normalized using 16S rRNA as an internal control.

### Statistical Analysis

All experiments were performed in triplicate and experimental data were presented as mean values ± standard deviation (SD). Statistical analysis was performed using Graphpad Prism 8 (San Diego, CA, United States). The data were analyzed with Student’s *t*-test and statistical significance was considered at *p < 0.05 and ^***^p < 0.01 for comparison.

## Results

### Identification of Phytochemical Compounds in *Corydalis turschaninovii* Rhizome Extract

The phytochemical compounds in the *C. turtschaninovii* rhizome extract were investigated by using HPLC-LTQ-Orbitrap-MS/MS, and 12 alkaloid compounds were identified based on their MS^1^ and MS^2^ fragmentation patterns (Supplementary Figures 1–3 and Supplementary Table 1). Based on the chemical structure, the identified alkaloids can be classified into four groups: protoberberine-, tetrahydroprotoberberine-, protopine-, and aporphine-alkaloids. Six protoberberine-alkaloids were identified, including coptisine (4), columbamine (7), berberine (8), dehydrocorybulbine (9), palmatine (10), and dehydrocorydaline (11). Four identified alkaloids belong to the tetrahydroprotoberberine-type, including corypalmine (1), yuanhunine (3) tetrahydropalmatine (5), and corydaline (6). The last two compounds, protopine (2) and oxoglaucine (12), were found to be alkaloids of the protopine- and aporphine-type, respectively.

### Antibacterial Activity of Rhizome Extract and Its Identified Compounds Against *Listeria monocytogenes*

**FIGURE 1:**
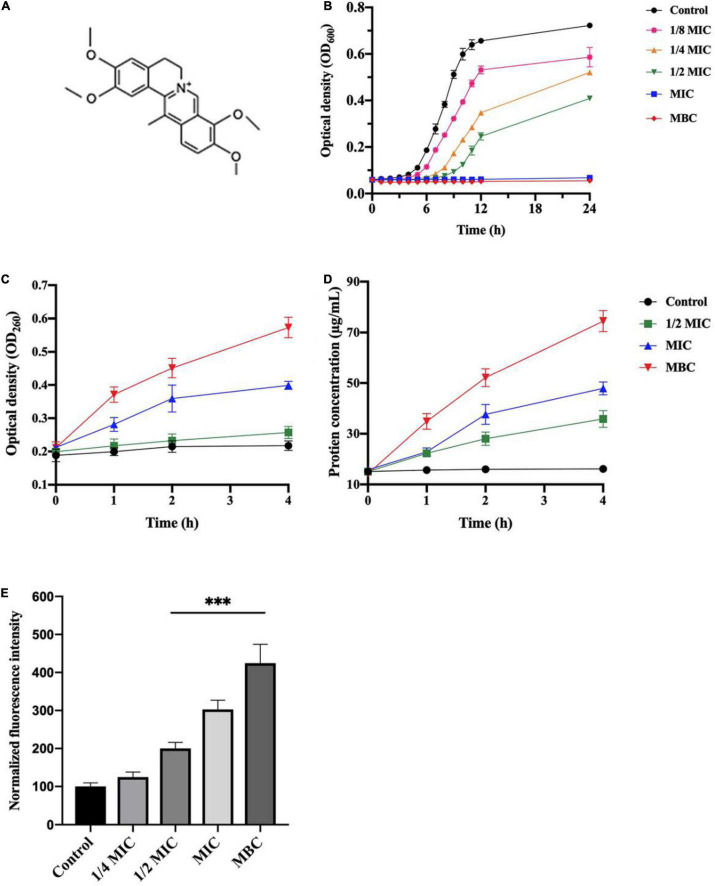
Antibacterial activity of dehydrocorydaline against *L. monocytogenes.* **(A)** Chemical structure of dehydrocorydaline, **(B)** growth curves of *L. monocytogenes*, **(C)** the nucleic acid, and **(D)** protein leakages of *L. monocytogenes* treated with various concentrations, and **(E)** ROS production in cells of *L. monocytogenes* treated with dehydrocorydaline. Data represent the mean ± S.D. ^***^Indicates *p* < 0.01 compared to the untreated group.

The antibacterial activity of the rhizome extract was evaluated by determining MIC and MBC, showing a strong antibacterial activity against *L. monocytogenes* with an MIC value of 3.125 mg/mL and MBC value of 6.25 mg/mL (Table 1). In order to identify major antibacterial compounds in the extract, 12 identified alkaloids were evaluated for bacteriostatic and bactericidal effects on *L. monocytogenes* (Table 1). Among constituents, dehydrocorydaline had the strong antibacterial effect (MIC = 1 mg/mL and MBC = 2 mg/mL). The MICs of columbamine and palmatine also had 1 mg/mL, but their MBCs were > 2 mg/mL. On the other hand, other compounds such as corypalmine, protopine, coptisine, tetrahydropalmatine, corydaline, berberine, dehydrocorybulbine, and oxoglaucine showed moderate activity (MIC = 2 mg/mL). Since dehydrocorydaline (Figure 1A) was a major component with strong antibacterial activity in the *C. turtschaninovii* rhizome extract, its effect on planktonic growth of *L. monocytogenes* was evaluated (Figure 1B). It showed good antibacterial effect *on L. monocytogenes* in a concentration-dependent manner. The MIC and MBC of dehydrocorydaline consistently inhibited the planktonic growth in 24 h.

**TABLE 1:** Determination of MIC (mg/mL) and MBC (mg/mL) values of *C. turtschaninovii* rhizome extract and identified compound.

	*L. monocytogenes* ATCC 7644		*S. aureus* ATCC 25923		*E. coli* ATCC 25922		*S. enterica* Enteritidis ATCC 13076	
	MIC	MBC	MIC	MBC	MIC	MBC	MIC	MBC
Rhizome extract	3.125	6.25	0.78	1.56	12.5	12.5	12.5	12.5
Corypalmine	2	>2	>2	ND	>2	ND	>2	ND
Protopine	2	>2	>2	ND	>2	ND	>2	ND
Yuanhunine	>2	ND^a^	>2	ND	>2	ND	>2	ND
Coptisine	2	>2	1	2	2	>2	>2	ND
Tetrahydropalmatine	2	>2	>2	ND	>2	ND	>2	ND
Corydaline	2	>2	>2	ND	>2	ND	>2	ND
Columbamine	1	>2	1	>2	1	2	2	>2
Berberine	1.5	>2	0.5	>2	1.5	>2	>2	ND
Dehydrocorybulbine	2	>2	1	>2	>2	ND	>2	ND
Palmatine	1	>2	1	2	1	>2	2	>2
Dehydrocorydaline	1	2	0.5	2	1	2	1	>2
Oxoglaucine	2	>2	2	>2	>2	ND	>2	ND
Ampicillin (μg/mL)	0.5	0.75	1	1.5	5	25	1	>25

*^a^ND, not determined.*

### Disruption of Cytoplasmic Membrane by Dehydrocorydaline

Leakage of cytoplasmic components, including nucleic acid and protein, was quantified to investigate the integrity of the bacterial cell membrane induced by dehydrocorydaline (Figures 1C,D). The results indicated that release of nucleic acid from *L. monocytogenes* increased in dose and time-dependent manners. Compared to untreated cells, a large amount of nucleic acid released from cells treated at MIC and MBC for 4 h were observed approximatively 1.8 times and 2.7 times, respectively (Figure 1C). Similarly, bacterial cells treated at MIC and MBC showed a large amount of protein leakage with time extension (Figure 1D).

**FIGURE 2:**
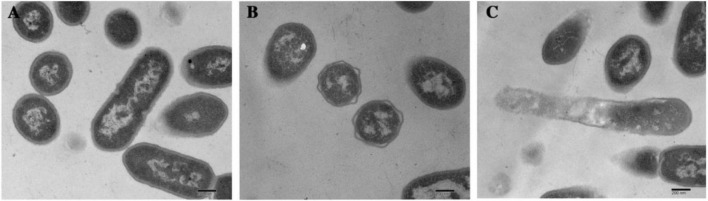
TEM analysis of *L. monocytogenes* cells treated with **(A)** 0, **(B)** MIC, and **(C)** MBC of dehydrocorydaline for 4 h, respectively.

Morphological and intracellular structural changes induced by dehydrocorydaline were also observed using Transmission Electron Microscopy (TEM). The *L. monocytogenes* cells were treated with or without dehydrocorydaline (Control, MIC, and MBC). As shown in Figure 2, the untreated cells had normal and uniform structure with complete organelles in the cytoplasm. On the other hand, the cells treated with dehydrocorydaline at MIC (1 mg/mL) showed irregularly shrunken membranes. In the case of cells treated at MBC (2 mg/mL), the cell walls disappeared, membranes were destroyed, and leakage of intracellular components was observed. These observations were consistent with the results of leakage of intracellular nucleic acid and protein, indicating that dehydrocorydaline somehow interfered with the integrity of the bacterial cell membrane.

### Dehydrocorydaline-Induced Reactive Oxygen Species Production

Intracellular ROS level was determined using a DCFH-DA assay to investigate the stimulation of ROS generation in *L. monocytogenes* by dehydrocorydaline. DCFH-DA can react with ROS (singlet oxygen, hydrogen peroxide, superoxide, and hydroxyl radical) generated from cell metabolism, and produce a fluorescent molecule DCF. As shown in Figure 1E, increased fluorescence intensity was displayed in *L. monocytogenes* treated with dehydrocorydaline, indicating that dehydrocorydaline induced ROS production in a dose dependent manner. Significant fluorescence intensity was detected at 1/2 MIC, MIC, and MBC of dehydrocorydaline, suggesting that excessive ROS production by dehydrocorydaline might contribute to bacterial cell death.

### Effect of Dehydrocorydaline on *Listeria monocytogenes* Biofilm Formation and Its Metabolic Activity

**FIGURE 3:**
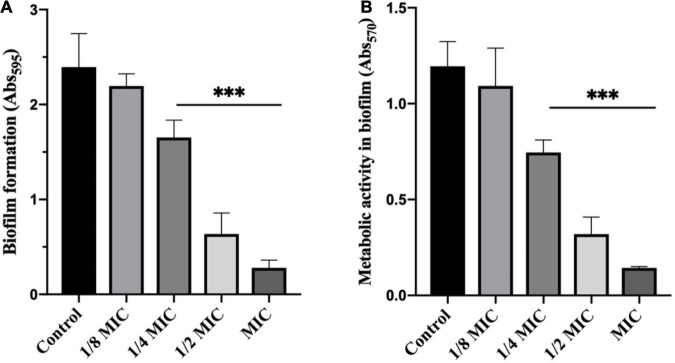
Antibiofilm effect of dehydrocorydaline on *L. monocytogenes*. **(A)** Inhibition of biofilm formation by sub-lethal concentrations, and **(B)** bacterial metabolic activity. ^***^Indicates *p* < 0.01 compared to untreated group.

In order to investigate the potential inhibition of dehydrocorydaline on biofilm formation, planktonic bacteria were treated with sublethal concentrations of dehydrocorydaline. The inhibitory effect was evaluated by quantification of biomass in biofilm and bacterial metabolic activity in biofilm. As shown in Figure 3A, dehydrocorydaline reduced biofilm formation in a concentration-dependent manner. Overall, 1/4 MIC, 1/2 MIC, and MIC of dehydrocorydaline significantly inhibited the initial biofilm formation compared to the control. In addition, bacterial metabolic activity in biofilm was determined using an MTT assay, showing that dehydrocorydaline at 1/4 MIC, 1/2 MIC, and MIC significantly inactivated bacterial metabolic activity in biofilms compared to the control (Figure 3B).

### Effect of Dehydrocorydaline on *Listeria monocytogenes* on Bacterial Motility

**FIGURE 4:**
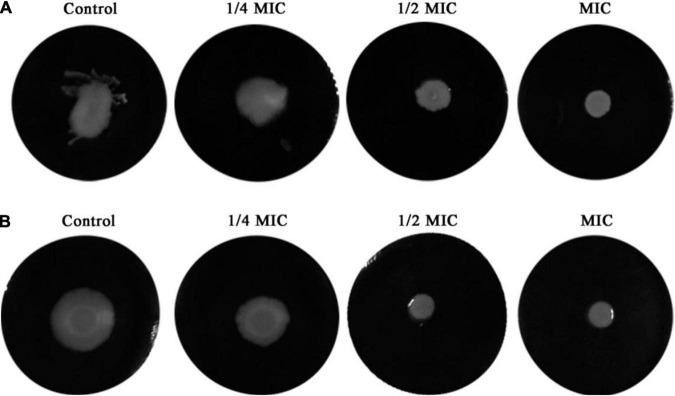
Inhibition of bacterial motility by sub-lethal concentrations of dehydrocorydaline. **(A)** Swimming motility and **(B)** swarming motility.

Inhibitory effects of dehydrocorydaline on *L. monocytogenes* cell motility, including swimming and swarming, were evaluated since bacterial motility is associated with initial biofilm formation (Oloketuyi and Khan, 2017). The results showed that dehydrocorydaline inhibited the swimming and swarming motility in a dose-dependent manner compared to the control (Figure 4). Dehydrocorydaline at 1/4 MIC, 1/2 MIC and MIC had good inhibitory effects on swimming motility (Figure 4A), while dehydrocorydaline at 1/2 MIC and MIC strongly inhibited the swarming motility (Figure 4B).

### Label-Free Quantitative Proteomic Analysis on *Listeria monocytogenes* in Response to Dehydrocorydaline

**FIGURE 5:**
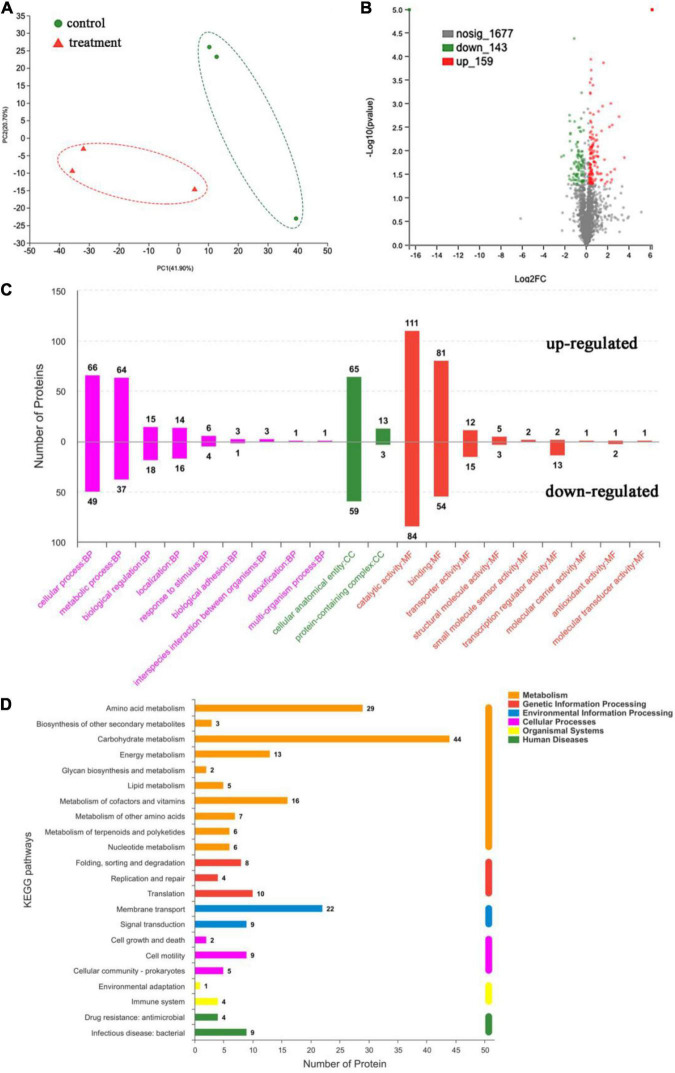
Label-free quantitative proteomics analysis. **(A)** Principal component analysis (PCA) of untreated *L. monocytogenes* cells (control) and cells treated with dehydrocorydaline. **(B)** Volcano plot of significantly differentially expressed proteins (DEPs) between the two group. **(C)** Gene Ontology (GO) enrichment analysis of DEPs, categorized into biological process (BP), cellular components (CC), and molecular function (MF). **(D)** Kyoto Encyclopedia of Genes and Genomes (KEGG) enrichment analysis of DEPs.

In order to investigate the specific mechanism of antibacterial action of dehydrocorydaline, label-free quantitative proteomic analysis was performed. Proteomics analysis identified a total of 1,924 proteins in control and treatment groups. As shown in Figure 5A, the PCA plot indicates that samples in each group were well separated. Proteins with an expression fold-change of ≥ 1.2 or ≤ 0.83 (*P* < 0.05) were considered as significantly differentially expressed proteins (DEPs), resulting in the identification of 444 DEPs in dehydrocorydaline-treated cells compared to untreated cells (Figure 5B). Among DEPs, 187 DEPs were downregulated in dehydrocorydaline-treated cells whereas 257 DEPs were upregulated.

#### Functional Classification of the Differentially Expressed Proteins

The DEPs were assigned to functional groups by Gene Ontology (GO) annotation to investigate their cellular functions in response to dehydrocorydaline treatment. As shown in Figure 5C, GO enrichment analysis was conducted to identify enriched DEPs and to classify them into three functional groups: cell components (CC), biological processes (BP), and molecular functions (MF). In the cell components, 124 DEPs were associated with cellular anatomical entity. In the biological processes, the cellular process and metabolic process had abundant DEPs (115 and 101 DEPs, respectively), followed by biological regulation (33 DEPs) and localization (30 DEPs). In the molecular functions, DEPs were mostly involved in catalytic activity (195 DEPs), followed by binding (135 DEPs), transporter activity (27 DEPs), and transcription regulator activity (15 DEPs).

#### Kyoto Encyclopedia of Genes and Genomes Analysis of Differentially Expressed Proteins

The DEPs were also assigned to KEGG pathway enrichment analysis to investigate the key biological pathways, in which DEPs are involved. As shown in Figure 5D, DEPs were mainly associated with metabolic pathways including carbohydrate metabolism (44 DEPs), amino acid metabolism (29 DEPs), cofactors and vitamin metabolism (16 DEPs), and energy metabolism pathways (13 DEPs).

Especially, in carbohydrate metabolism, 11 proteins responsible for fructose and mannose metabolism, 10 proteins for amino sugar and nucleotide sugar metabolism, 8 proteins for glycolysis, 7 proteins for starch & sucrose metabolism, and 6 proteins for pyruvate metabolism were changed by dehydrocorydaline treatment (Table 2). These metabolism-related proteins are closely associated with the membrane transporters (22 DEPs) such as ABC transporters and phosphotransferase system (PTS). To be specific, the carbohydrate metabolisms in cells treated with dehydrocorydaline were regulated by differently expressed transporter proteins (ManZ, ManXa, CmtB, FruK, GfrB, CelA, CelC, GatA, and GatC) involved in phosphoenolpyruvate (PEP): carbohydrate PTS. Consistent with these regulations, DEPs (GltA, PdhD, Pyc, and FrdA) involved in a tricarboxylic acid (TCA) cycle were changed. In addition, several downregulated proteins involved in glycolysis, glycogenesis, and the PP pathway were identified: glucose-6-phosphate isomerase (GPI), manose-6-phosphate isomerase (ManA), ribose-5-phosphate isomerase (RpiA), and galactose mutarotase (GalM). The ROK family protein (ScrK), which is a transcriptional regulator, was also suppressed.

**TABLE 2:** Representative differentially expressed proteins (DEPs) of *L. monocytogenes* treated with dehydrocorydaline.

UniProt accession no.	Description	Gene	Fold change
**Membrane transporter**			
A0A6W6TJD5	PTS mannose family transporter subunit IID	*manZ*	0.5595↓
A0A6W7DRZ2	PTS mannose transporter subunit IIAB	*manXa*	0.3126↓
A0A6Z3R5N5	PTS mannose transporter subunit IIA	*manXa*	0.5513↓
A0A0B8QR16	PTS sugar transporter subunit IIA	*cmtB*	0.6582↓
A0A608GCL0	1-phosphofructokinase family hexose kinase	*fruK*	0.3851↓
A0A3Q0NG66	PTS sugar transporter subunit IIB	*gfrB*	0.4389↓
A0A6V9TPP0	PTS sugar transporter subunit IIB	*celA, chbB*	0.1891↓
A0A700N3D0	PTS lactose/cellobiose transporter subunit IIA	*celC, chbA*	0.204↓
A0A6Z1WA42	PTS galactitol transporter subunit IIC	*gatC, sgcC*	2.634 ↑
A0A6W9K6Y7	PTS sugar transporter subunit IIA	*gatA, sgcA*	3.34↑
A0A6Y7WF20	ATP-binding cassette domain-containing protein	*mdlA, smdA*	0.4232↓
A0A0E1R7F3	Putative ABC transporter ATP-binding protein exp8	*mdlB, smdB*	0.5283↓
A0A6X2HDQ7	ABC transporter permease	*tagG*	0.7352↓
**Metabolism**			
A0A6W9FKC9	Glucose-6-phosphate isomerase	*gpi*	0.121↓
A0A6Z2VBL9	Mannose-6-phosphate isomerase	*manA*	0.301↓
A0A6Z0SRA5	Ribose-5-phosphate isomerase RpiA	*rpiA*	0.748↓
A0A6X5TZI5	Galactose mutarotase	*galM*	0.4123↓
A0A3T2ENW3	ROK family protein	*scrK*	0.8173↓
A0A6W4L1A5	Pyruvate carboxylase	*pyc*	1.354↑
A0A6X3YNN0	Citrate synthase	*gltA*	2.567 ↑
A0A6W3U286	Dihydrolipoyl dehydrogenase	*pdhD*	1.59↑
A0A6Z3KQR8	Flavocytochrome c	*frdA*	0.7295↓
**Cell wall synthesis**			
A0A462UTU4	Glutamine-fructose-6-phosphate transaminase	*glmS*	0.7568↓
A0A6X2V2U3	Undecaprenyldiphospho-muramoylpentapeptide ß-N-acetylglucosaminyltransferase	*murG*	0.7812↓
**Bacterial motility**			
A0A241SNH8	Flagellar motor switch protein FliG	*fliG*	0.6623↓
Q5Y832	FliC/FljB family flagellin	*fliC*	0.5059↓
Q8Y933	Flagellar protein FliS	*fliS*	0.59↓
A0A6X2UYD9	Flagellar hook-associated protein 2	*fliD*	0.5348↓
A0A0H3GA16	Flagellar hook-associated protein 3	*flgL*	0.6233↓
A0A0E1Y5K1	Flagellar hook-basal body complex protein	*fliE*	0.112↓
A0A6Y8N079	Flagellar basal body M-ring protein	*fliF*	0.5973↓

*Listeria monocytogenes* treated with dehydrocorydaline also altered the expression of two DEPs that participate in cell wall synthesis: glutamine-fructose-6-phosphate transaminase (GlmS) and undecaprenyldiphospho-muramoylpentapeptide β-N-acetylglucosaminyltransferase (MurG), with fold changes of 0.757 and 0.781, respectively (Table 2).

In terms of cellular processes, the expression of flagellar assembly proteins including hook-filament junction proteins (FlgL), flagellar filament proteins (FliC, FliD, and FliS), and basal body proteins (FliE, FliF, and FliG) were downregulated by dehydrocorydaline.

### Validation of Label-Free Quantitative Proteomic Analysis Results at the mRNA Level

**FIGURE 6:**
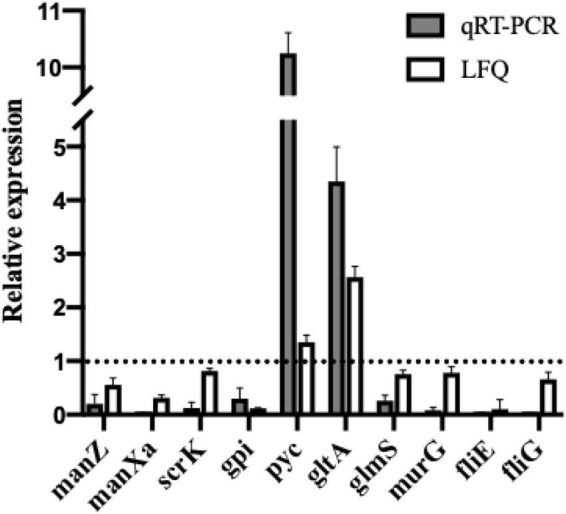
Comparison of relative quantitation results between qRT-PCR and label-free proteomics (LFQ). Relative expression levels of *manZ, manXa, scrK, gpi, pyc, gltA, glmS, murG, fliE*, and *fliG* in *L. monocytogenes* treated with dehydrocorydaline were determined by qRT-PCR to verify proteomics results. The 16S rRNA gene was used as the housekeeping gene.

To validate the proteomic results, real-time PCR was conducted to analyze mRNA transcription levels of the selected DEPs, including ManZ, ManXa, GPI, ScrK, Pyc, GltA, GlmS, MurG, FliE, and FliG. As shown in Figure 6, the mRNA expression levels of selected proteins by qRT-PCR showed consistent expression patterns with label-free quantitative proteomics results (LFQ), demonstrating the reliability of the proteomics data.

## Discussion

In recent years, there are urgent demands for development of new strategies to combat *L. monocytogenes* due to the emergence of antibiotic-resistant bacteria and a failure of antibiotic treatment, causing the gradually increasing incidence of listeriosis (Radoshevich and Cossart, 2018). One such strategy is the application of natural antibacterial compounds that have gained attention as alternatives due to its safety and antibacterial efficacy (Silva et al., 2016). Phytochemical compounds are able to inactivate the bacterial growth by targeting multiple sites such as cellular mechanism, disturbance of cell membrane, and modulation of virulence factors, which can effectively combat antibiotic-resistant bacteria (Silva et al., 2016).

The *C. turtschaninovii* rhizome extract contains various natural isoquinoline alkaloids, which consist of nitrogen containing a heterocyclic structure (a benzene ring fused to a pyridine ring). This unique structural feature of *C. turtschaninovii* is responsible for distinctive bioactivities including analgesic (Zhang et al., 2014), anti-amnesic (Hung et al., 2008), anti-inflammatory (An et al., 2009), and anti-cancer effects (Kim et al., 2012; Lee et al., 2017). Although the bioactivities of *C. turtschaninovii* have been studied, its antibacterial effect still remains unknown. Our preliminary study demonstrated that the rhizome of *C. turtschaninovii* exhibited potent antibacterial properties (Kim et al., 2020), thereby further studying its antibacterial properties against *L. monocytogenes* is important. In order to identify major antibacterial compounds in the extract, 12 identified alkaloids were evaluated for bacteriostatic and bactericidal effects on *L. monocytogenes*, indicating that all compounds except yuanhunine had strong antibacterial effects and dehydrocorydaline exhibited the greatest (MIC = 1 mg/mL and MBC = 2 mg/mL) (Table 1). Comparable results for protopine, berberine, coptisine, palmatine, and columbamine were previously reported from other studies, demonstrating that these isoquinoline alkaloids have broad-spectrum antibacterial activity against *Bacillus subtilis, Brucella abortus, Escherichia coli, Helicobacter pylori, Staphylococcus aureus*, and *Salmonella enterica* Enteritidis (Iwasa et al., 1998; Mahady et al., 2003; Cheng et al., 2014; Azimi et al., 2018). Liu et al. (2015) reported that berberine exhibited good antibacterial activity against *L. monocytogenes* (MIC = 8 mg/mL). However, unlike other isoquinoline alkaloids, the antibacterial activity of dehydrocorydaline has not been reported. Interestingly, it is also found that the protoberberine-type of alkaloids, including berberine, columbamine, coptisine, dehydrocorybulbine, dehydrocorydaline and palmatine, exhibited stronger antibacterial effect than other types of alkaloids. This high effectiveness might be attributed to the presence of a free hydroxyl group on the C-2 or C-3 position (Long et al., 2019), dioxymethylene at C-2 and C-3 positions on the ring (Xiao et al., 2018), or an alkyl group containing a pyridine ring (Iwasa et al., 1997).

To the best of our knowledge, this study reports the antibacterial activity of dehydrocorydaline for the first time. Since dehydrocorydaline was a major component with the strongest antibacterial activity among the *C. turtschaninovii* constituents, it was used to further study its potential antibacterial mechanisms of action against *L. monocytogenes*. Isoquinoline alkaloids have been studied for their antibacterial mechanisms with respect to their distinctive chemical structures such as inhibition of bacterial nucleic acid synthesis and metabolism, interference with cell membrane transporters, suppression of cell division, and disruption of cell membrane, leading to bacterial cell lysis (Cushnie et al., 2014; Yan et al., 2021). These studies agree with our results, indicating that *L. monocytogenes* cells treated with dehydrocorydaline showed morphological and intracellular structural changes such as disappearance of cell walls, membrane destruction, and leakage of intracellular components in TEM observation (Figure 2). Also, the large amounts of nucleic acid and protein leaking were observed in bacterial cells treated with dehydrocorydaline compared to untreated cells (Figures 1C,D). These phenomena indicated that dehydrocorydaline somehow interfered with the integrity of the bacterial cell membrane.

**FIGURE 7:**
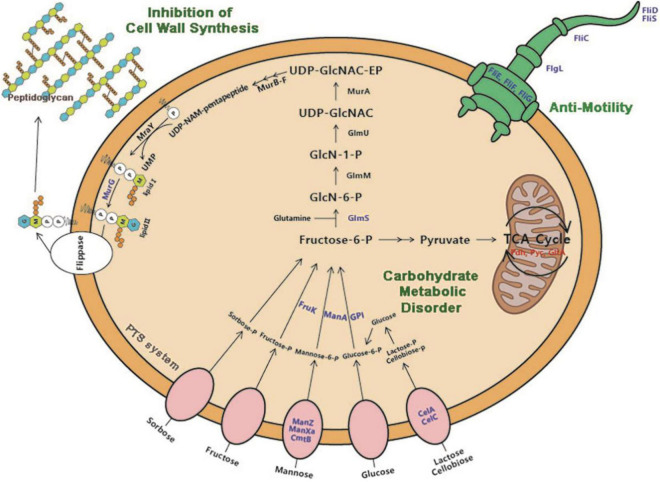
Proposed antibacterial mechanisms of action of dehydrocorydaline on *L. monocytogenes*: carbohydrate metabolic disorder, suppression of cell wall synthesis, and inhibition of bacterial motility. Downregulated protein by dehydrocorydaline is in blue color and upregulated protein is in red color.

At present, proteomic technologies have been widely applied to understand the antibacterial action mode of antimicrobial agents against pathogens for drug discovery (Ma et al., 2017). Herein, quantitative proteomic analysis was performed to investigate the molecular mechanism of antibacterial action of dehydrocorydaline against *L. monocytogenes*. Taken together with proteomic results, we found that dehydrocorydaline influenced the global proteomic alteration of *L. monocytogenes* associated with carbohydrate metabolism, energy metabolism, cell wall synthesis, and bacterial motility, suggesting multiple target sites of dehydrocorydaline to inactivate bacterial growth (Figure 7).

Especially, dehydrocorydaline significantly altered the expression of *L. monocytogenes* proteins associated with carbohydrate metabolism, energy metabolism, and membrane transporters such as ABC transporters and PTS (Figure 5D and Table 2). Targeting bacterial central metabolism, especially carbohydrate metabolism, has been suggested for a novel strategy to combat antibiotic-resistant bacteria, thanks to the presence of unique bacterial enzymes and hypersensitivity to antibacterial agents by changing their metabolism (Murima et al., 2014). In carbohydrate metabolism, the main pathways include the PTS, glycolysis, gluconeogenesis, pentose phosphate (PP) pathway, and a TCA cycle (Papagianni, 2012). The bacterial PTS, only present in bacteria, is responsible to transport and catalyze the phosphorylation of extracellular carbohydrates that provide various carbon sources for bacterial metabolism (Deutscher et al., 2020). The PTS are comprised of two cytoplasmic energy coupling proteins [Enzyme I (EI) and phosphocarrier protein (HPr)] and various carbohydrate specific enzymes II (EIIA, EIIB, and EIIC). The extracellular carbohydrates bind to EIIC, transport to the cytoplasm, and convert to carbohydrate-phosphate (P) by a phosphoryl transfer from phosphoenol-pyruvate (PEP) via EI, HPr, EIIA, and EIIB, successively. Then, intercellular carbohydrate-P catalyze into fructose 6-P via several interconnected pathways for metabolism (Kotrba et al., 2001). In bacterial cells treated with dehydrocorydaline, various carbohydrate PTS components such as EIIC, EIIB, and EIIA were downregulated: IIA*^man^*, IIB*^man^*, IID*^man^*, IIB*^fru^*, and IIB*^sor^* in mannose-fructose-sorbose family specific PTS, and IIA*^Lic^* and IIB*^Lic^* in lactose-cellobiose family specific PTS. This result suggests that bacterial cells lack access to carbohydrate sources, which are transported by a phosphate-dependent PTS system. Consequently, other proteins (GPI, ManA, RpiA, GalM, ScrK, GltA, PdhD, Pyc, and FrdA) involved in glycolysis, glycogenesis, PP pathway, and a TCA cycle were also changed, leading to the alteration of metabolic state of *L. monocytogenes* cells, especially carbon metabolic dysregulation. In consequence of this carbohydrate metabolic dysregulation, *L. monocytogenes* lack fructose-6-P as an initial metabolite used in many metabolisms that result in the unbalance of energy metabolism and DNA, protein, and lipid biosynthesis (Du et al., 2020). The reduction of carbohydrate metabolic activity can contribute to the accumulation of unused intercellular ATP, NADP, and metabolites, triggering the disruption of bacterial homeostasis, the stimulation of excessive ROS, and cell death (Stokes et al., 2019). This result was verified by an RT-PCR assay and the measurement of ROS, showing that dehydrocorydaline induced carbohydrate metabolic dysfunction (upregulated *pdh*, *pyc*, and *gltA*), and caused an increase in the intercellular ROS level as a by-product of metabolism (Figure 1E). Previous study conducted by Du et al. (2020) demonstrated that berberine, a protoberberine-type alkaloid like dehydrocorydaline, caused carbohydrate metabolic disorders in *Streptococcus pyogenes*, consistent with our study. Contrary to downregulation of the PTS system and upregulation of glycolysis, glycogenesis, the PP pathway, and a TCA cycle in our study, berberine promoted the uptake and conversion of carbohydrate by the PTS system, but suppressed the phosphofructokinase involved in glycolysis.

*L. monocytogenes*, a Gram-positive bacteria, is encased by thick layers of peptidoglycan, a covalently cross-linked polymer matrix composed of peptide-linked β-(1-4)-*N*-acetyl hexosamine (Kohanski et al., 2010). The layers of cell wall play an important role to maintain cell shape, to resist osmotic pressure from the cytoplasm, and to protect from the stresses of the external environment (Kohanski et al., 2010). In the past decade, the peptidoglycan (PG) biosynthetic pathway has been intensively studied as effective target sites to develop a new class of antibacterial agents which are vital for bacterial survival and have no counterpart in mammalian cells (Kouidmi et al., 2014). According to proteomics results, dehydrocorydaline downregulated GlmS and MurG involved in cell wall synthesis of *L. monocytogenes*, leading to the inhibition of cell wall synthesis. This is possibly associated with the downregulation of PTS proteins by dehydrocorydaline. In PG wall synthesis, fructose-6-phophate, which is transported and phosphorylated by the PTS, is essential as a primary precursor for Glm enzymes (GlmS, GlmM, and GlmU) to be converted with glutamine into UDP-GlcNAc (Bearne, 1996; Liu and Breukink, 2016) (Figure 7). GlmS is the prior enzyme to catalyze GlcN-6-phosphate from fructose-6-phosphate and glutamine. On the other hand, unlike other Mur enzymes (MurA, MurB, MurC, MurD, MurE, and MurF) that convert UDP-*N*-acetylglucosamine into UDP-*N*-acetylmuramoly-pentapeptide, MurG plays a distinctive role at the cytoplasmic membrane (Nikolaidis et al., 2014). After phospho-MurNAc-pentapeptide translocase (MraY) generates a polyprenyl-linked precursor, called undecaprenyl-pyrophosphoryl-MurNAc-pentapeptide (lipid I), MurG subsequently catalyzes the transfer of the GlcNAc moiety from UDP-GlcNAc to lipid I, producing undecaprenyl-pyrophosphoryl-MurNAc-(pentapeptide)-GlcNAc (lipid II), which is then transported by a flippase from cytoplasmic membrane to periplasm wherein the polymerization of peptidoglycan happens (Müller et al., 2017). Due to the deficiency of carbon sources transported by PTS, *L. monocytogenes* treated with dehydrocorydaline utilize carbon sources toward the central metabolism rather than anabolism such as cell wall synthesis, likely contributing to the suppression of GlmS and MurG (Table 2). These suppressed proteins could cause the inhibition of PG synthesis, destruction of the peptidoglycan layer, loss of integrity, and thereby cell death. In the present study, we provided SEM observations (Figure 2) and the quantification of leakage of intercellular components such as nucleic acids and proteins (Figures 1C,D) to support the loss of bacterial cell integrity induced by dehydrocorydaline. Moreover, the stimulation of carbon flux toward the central metabolism in *L. monocytogenes* treated with dehydrocorydaline might produce excessive ROS through the TCA cycle (upregulation of Pdh, Pyc, and GltA in Figure 7 and Table 2), triggering cell death. This tendency was observed by Kawai et al. (2019), showing that the suppression of cell wall synthesis results in the increase of carbon flux toward catabolic activity, the stimulation of the TCA cycle, and the abnormally increased ROS levels.

Bacterial flagellum and its motility are virulence factors in charge of attachment to biotic or abiotic surfaces, colonization, and biofilm formation, resulting in survival in a hostile environment and resistance to antibacterial agents by not allowing their penetration into the bacterial biofilm (Xin et al., 2019). We demonstrated that dehydrocorydaline at sub-MICs can prevent initial biofilm formation and biofilm development of *L. monocytogenes*, and also reduce biofilm metabolic activity (Figure 3). The effects of dehydrocorydaline are probably associated with the inhibition of flagella formation and flagellum-mediated motility. Two types of *L. monocytogenes* cell motility, swimming and swarming, were present (Renier et al., 2010). Swimming motility, which is individual movement by rotating flagella in liquid, is involved in the initial migration and adherence to a surface. Swarming motility is multicellular surface movement and contributes to cellular aggregates for biofilm formation (Thakur et al., 2020). In this study, dehydrocorydaline hindered swimming and swarming motility at sublethal concentrations (Figure 4), contributing to inhibition in the early stages of biofilm formation. The corresponding results revealed by proteomic analysis indicated that dehydrocorydaline downregulated the expression of flagellar assembly proteins (FlgL, FliC, FliD, FliS, FliE, and FliF) and flagellar motor protein (FliG).

## Conclusion

In summary, we identified 12 alkaloid compounds in *C. turtschaninovii* rhizome extract and evaluated the antibacterial activity of the identified compounds, showing that dehydrocorydaline has a strong antibacterial effect on *L. monocytogenes*. To the best of our knowledge, this is first report of dehydrocorydaline as a potential antibacterial agent. To investigate potential antibacterial mechanisms of action of dehydrocorydaline, label-free quantitative proteomic analysis was performed. From the perspective of proteomics, dehydrocorydaline has multiple targets for combating *L. monocytogenes* such as the dysregulation of carbohydrate metabolism, the suppression of cell wall synthesis, and the inhibition of bacterial motility. Our findings suggest dehydrocorydaline is a natural and effective agent with multiple antibacterial targets, providing the potential possibility for development of a new class of antibacterial agent to combat antibiotic resistance.

## Data Availability Statement

The datasets presented in this study can be found in online repositories. The name of the repository/repositories and accession number are IProX, IPX0003626000 in the article/Supplementary Material.

## Author Contributions

GK performed experiments, analyzed data, and wrote the manuscript. YX and JZ analyzed data and edited the manuscript. ZS supervised the project and edited the manuscript. HC acquired funding, supervised the project, and edited the manuscript. All authors contributed to the article and approved the submitted version.

## Conflict of Interest

The authors declare that the research was conducted in the absence of any commercial or financial relationships that could be construed as a potential conflict of interest.

## Publisher’s Note

All claims expressed in this article are solely those of the authors and do not necessarily represent those of their affiliated organizations, or those of the publisher, the editors and the reviewers. Any product that may be evaluated in this article, or claim that may be made by its manufacturer, is not guaranteed or endorsed by the publisher.

## Footnotes

1
http://www.geneontology.org/

2
http://www.genome.jp/kegg/
