# Supplementary material for: Antibacterial Activity and Multi-Targeting Mechanism of Dehydrocorydaline From Corydalis turtschaninovii Bess. Against Listeria monocytogenes
Source: Front Microbiol. 2022 Jan 11;12:799094. doi: 10.3389/fmicb.2021.799094 (PMC8787222; doi:10.3389/fmicb.2021.799094)
Supplement: Supplementary file 1 [file Data_Sheet_1.docx]

**Supplementary material**

**Antibacterial activity and multi-targeting mechanism of dehydrocorydaline from Corydalis turtschaninovii Bess. against Listeria monocytogenes**

**Gowoon Kim^1^, Yijuan Xu^1^, Jiarong Zhang^2^, Zhongquan Sui^1*^ and Harold Corke^2,3*^**

^1^Department of Food Science and Technology, Shanghai Jiao Tong University, Shanghai 200240, China

^2^Biotechnology and Food Engineering Program, Guangdong Technion – Israel Institute of Technology, Shantou 515063, China

^3^Faculty of Biotechnology and Food Engineering, Technion-Israel Institute of Technology, Haifa 3200003, Israel

*Correspondences:

Zhongquan Sui

zsui@sjtu.edu.cn.

Harold Corke

harold.corke@gtiit.edu.cn.

**Supplementary Table 1.** Characterization of the alkaloid constituents in ethanolic extract of *C. turtschaninovii* rhizome using HPLC-LTQ-Orbitrap-MS/MS and evaluation of their antibacterial activity against *L. monocytogenes* ATCC 7644.

| Peak | $t_{R}$ (min) | formula | MS^1^ (*m/z*) | MS^2^ (*m/z*) | compound |
| --- | --- | --- | --- | --- | --- |
|  |  |  |  |  |  |
| 1*^a^* | 16.30 | C_20_H_23_NO_4_ | 342.25  ${[M+H]}^{+}$ | 311.24 (100), 221.11 (33), 192.15 (17), 178.12 (87) | corypalmine |
| 2*^a^* | 18.28 | C_20_H19NO_5_ | 354.20  ${[M+H]}^{+}$ | 336.22 (89), 323.21(42), 305.14 (27), 275.15 (40), 206.04 (37), 190.11 (83), 189.87(37), 188.11(100), 149.09 (64), 119.10 (12) | protopine |
| 3*^a^* | 21.13 | C_21_H_25_NO_4_ | 356.20  ${[M+H]}^{+}$ | 325.18 (100), 192.04 (8), 165.08 (6) | yuanhunine |
| 4*^a^* | 21.77 | C_19_H_14_NO_4_ | 320.16  $[{M]}^{+}$ | 318.24 (17), 293.15 (13), 292.07 (100) | coptisine |
| 5*^a^* | 23.34 | C_21_H_25_NO_4_ | 356.23  ${[M+H]}^{+}$ | 340.35 (9), 324.40 (11), 300.51 (10), 273.47 (14), 204.14 (6), 192.10 (100), 191.20 (34), 190.08 (37), 165.13 (51), 150.07(9) | tetrahydropalmatine |
| 6*^a^* | 24.18 | C_22_H_27_NO_4_ | 370.23  ${[M+H]}^{+}$ | 339.35 (24), 325.25 (100), 324.26 (19), 301.32 (12), 218.18 (16), 205.98 (8), 192.15 (70), 190.24 (15), 179.12 (14), 165.20 (57), 150.03 (13) | corydaline |
| 7*^a^* | 25.61 | C_20_H_20_NO_4_ | 338.22  $[{M]}^{+}$ | 324.27 (18), 323.22 (100), 322.14 (12), 294.15 (12), 255.93 (2), 241.12 (1), 190.09 (1), 177.95 (2) | columbamine |
| 8*^a^* | 27.93 | C_20_H_18_NO_4_ | 336.25  $[{M]}^{+}$ | 334.27 (17), 321.16 (100), 309.16 (33), 292.15 (18), 278.38 (2), 176.15 (1) | berberine |
| 9*^b^* | 28.53 | C_21_H_22_NO_4_ | 352.24  $[{M]}^{+}$ | 338.21 (23), 337.23 (100), 336.20 (12), 308.23 (10), 278.14 (1), 271.16(2), 264.25(1) | dehydrocorybulbine |
| 10*^a^* | 29.02 | C_21_H_22_NO_4_ | 352.26  $[{M]}^{+}$ | 337.21 (100), 308.24 (13), 271.21(4), 206.00 (1), 190.13(1) | palmatine |
| 11*^a^* | 31.35 | C_22_H_24_NO_4_ | 366.24  $[{M]}^{+}$ | 352.34 (21), 351.23 (100), 336.21 (32), 318.17 (8) | dehydrocorydaline |
| 12*^a^* | 61.72 | C_20_H_17_NO_5_ | 352.21  ${[M+H]}^{+}$ | 344.64 (10), 342.75 (24), 338.30 (30), 337.25 (100), 322.12 (18), 307.21 (25), 306.21(92), 286.22(3), 262.57 (2), 239.12 (2), 230.07 (4) | oxoglaucine |

*^a^*Confirmed by authentic standard. *^b^*Confirmed by reference (Sun et al., 2014; Wang et al., 2017). *^e^*ND, not determined.

**Supplementary Table 2.** List of primers used for qRT-PCR

| Genes | Forward primer ($5'\to3'$) | Reverse primer ($5'\to3'$) |
| --- | --- | --- |
| *fliE* | GGTGAAACCGCGAAAACAGA | TCGCATTTCCTTCGCCAGTT |
| *fliG* | CGCAGTGGAAGAAGCGACTA | CGTCGTCATGTCTGGGAACA |
| *glmS* | CAGACCTTGCGAGCCTTGTA | GGCGTTTTCGTGATTCGGTT |
| *gltA* | GAGTTTGTGTGGCAACGCTT | ACGTTCATTAGCACCACCGT |
| *gpi* | CAGACCTTGCGAGCCTTGTA | GGCGTTTTCGTGATTCGGTT |
| *manXa* | CAATGGAAAGCGCACACGAA | GCGGCTGTAGTTCTTCTGGT |
| *manZ* | ATGGCGATTGAAGAGCGAGA | ATCACCAACACCAGCAAACG |
| *murG* | AGCAGGAGCGACTACACTTG | GGTTCGCTGTCACGTATGGA |
| *pyc* | TCGTTGTGAGCAAGAAGGCA | CAGCCACGGGTCCATTACTT |
| *scrK* | ATCGGAACAGGAATCGGTGG | CTCGGGCAACTTCCTGTGAA |
| 16s RNA | ACCGTCAAGGGACAAGCA | GGGAGGCAGCAGTAGGGA |

**Supplementary Figure 1.** HPLC chromatogram of ethanolic extract of *C. turtschaninovii* rhizome


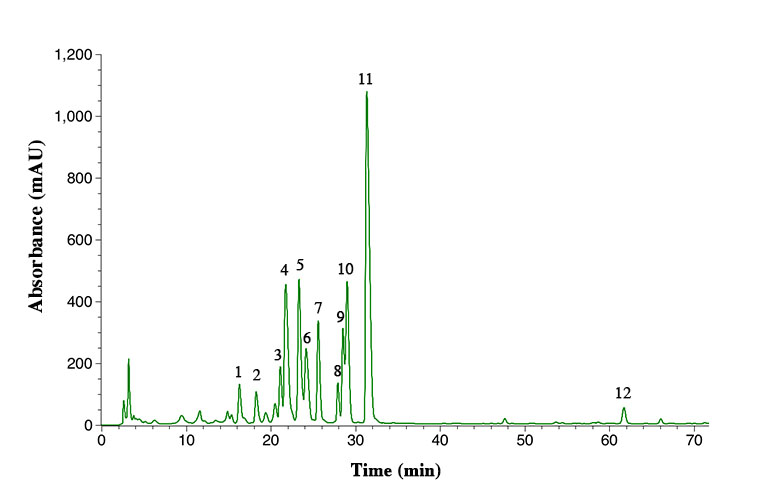


**Supplementary Figure 2.** LC-MS total ion chromatogram of *C. turtschaninovii* tuber extract in positive (A) and negative (B) ion modes.

1. MS_positive mode


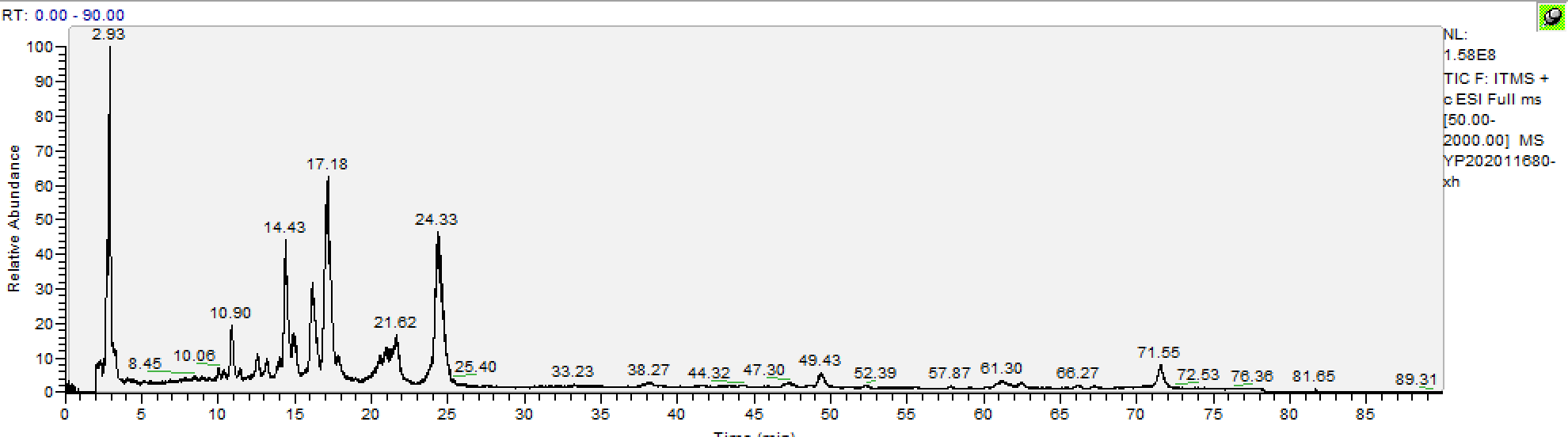


1. MS_negative mode


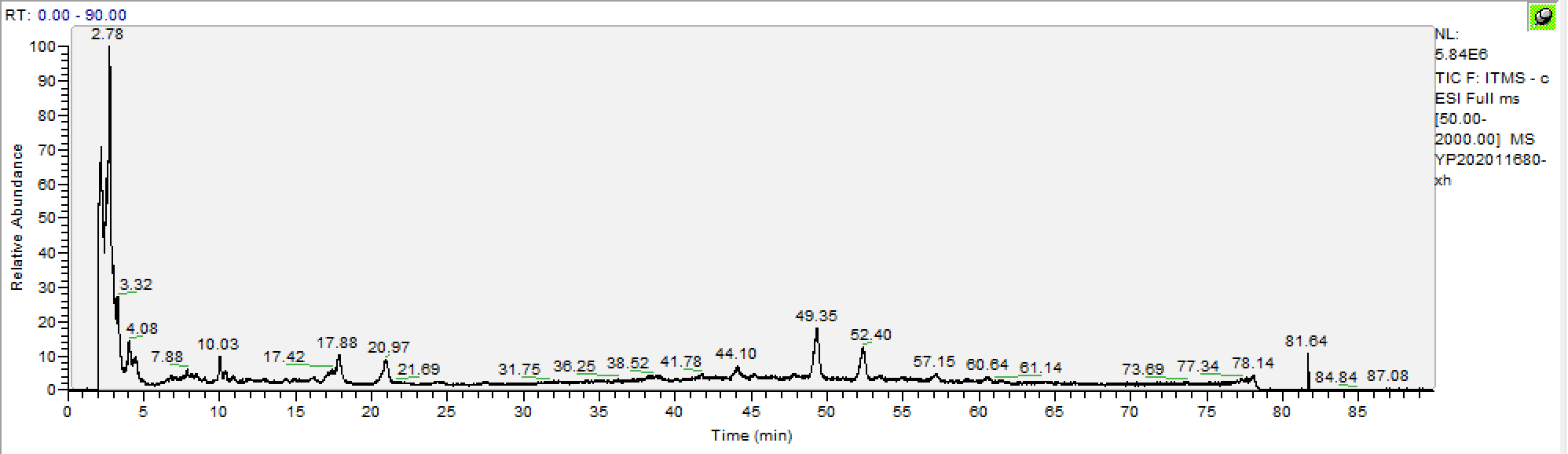


**Supplementary Figure 3.** MS^1^ and MS^2^ spectra of compounds 1-12

**Compound 1:** corypalmine

MS^1^


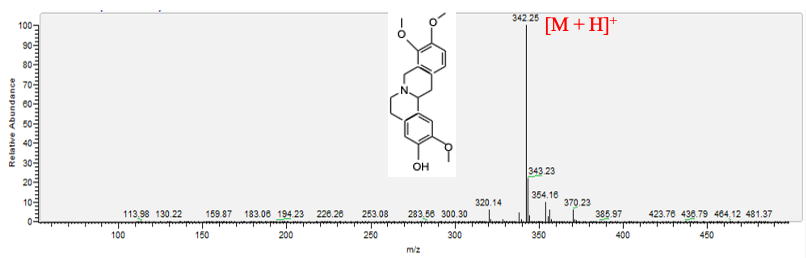


MS^2^

**
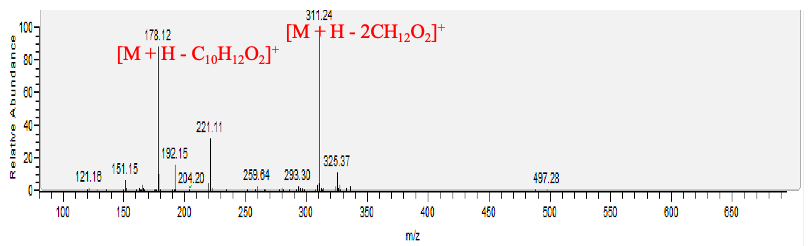
**

**Compound 2:** protopine

MS^1^

^
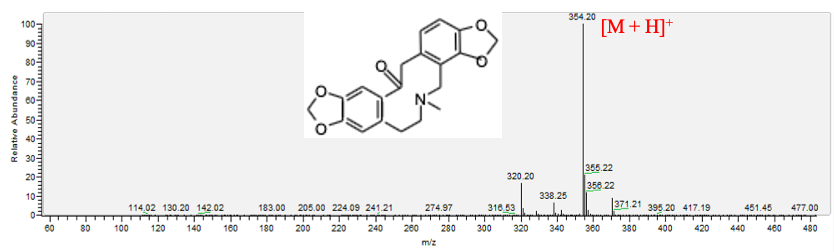
^

MS^2^

**
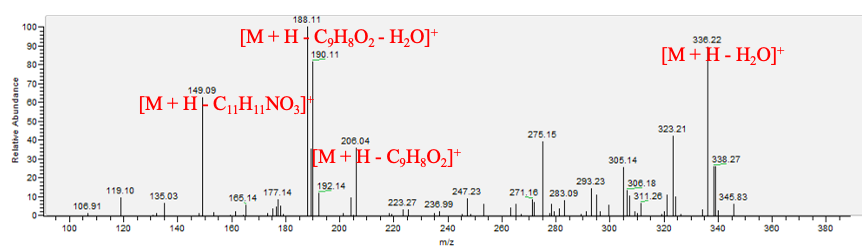
**

**Compound 3:** yuanhunine

MS^1^


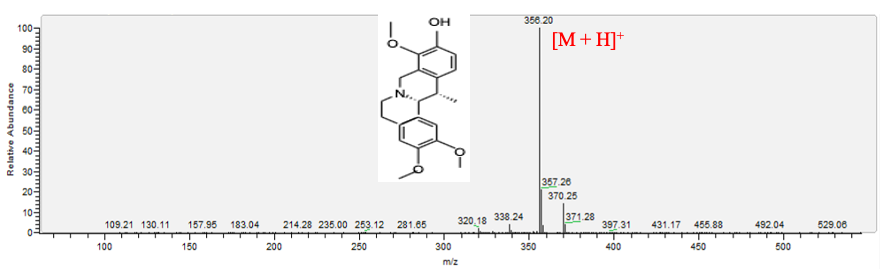


MS^2^

**
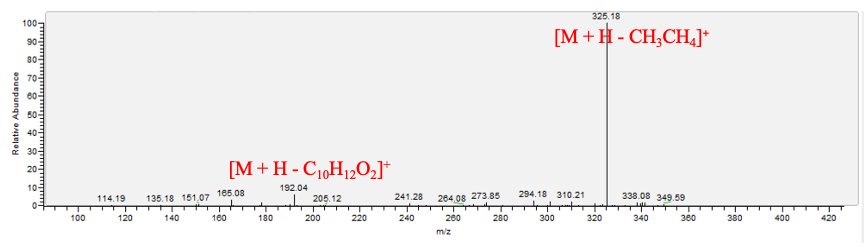
**

**Compound 4:** coptisine

MS^1^

^
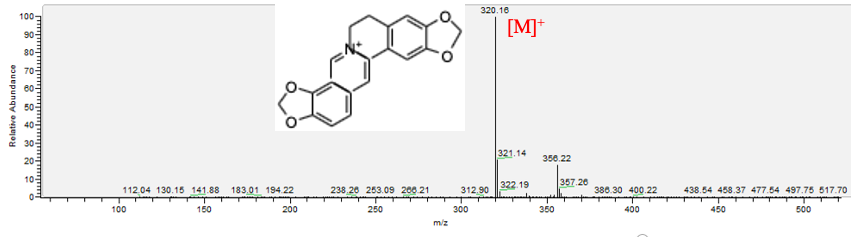
^

MS^2^

**
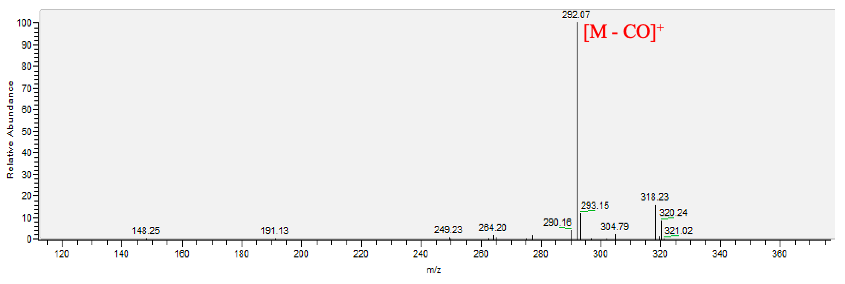
**

**Compound 5:** tetrahydropalmatine

MS^1^


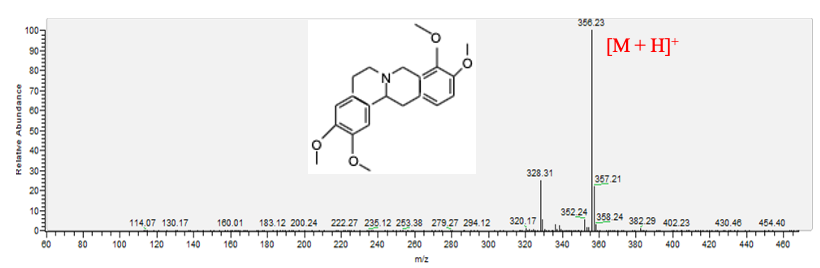


MS^2^

**
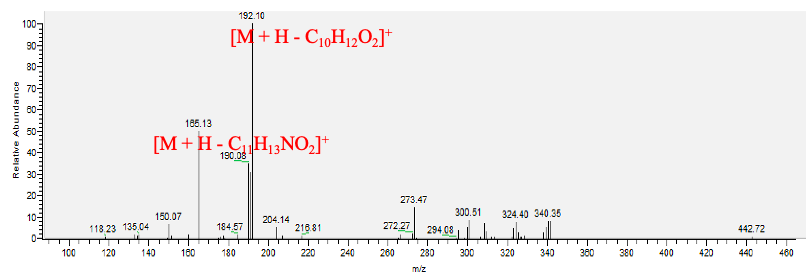
**

**Compound 6:** corydaline

MS^1^


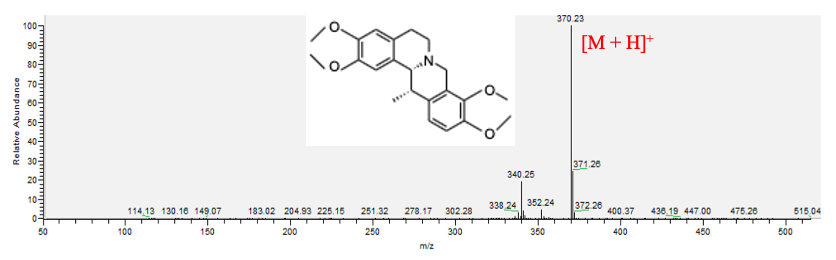


MS^2^

**
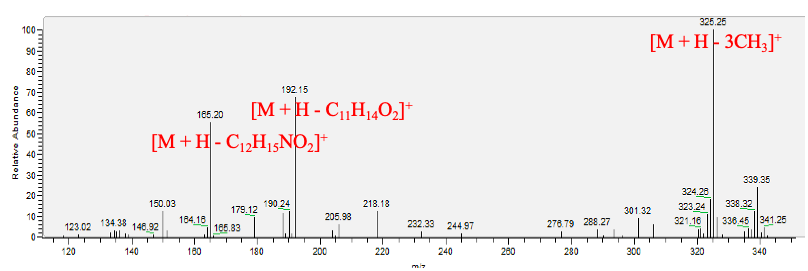
**

**Compound 7:** columbamine

MS^1^

^
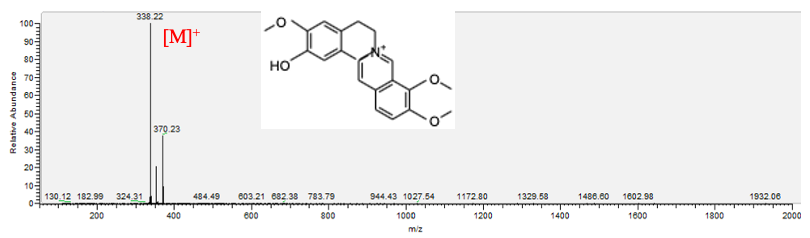
^

MS^2^ ^
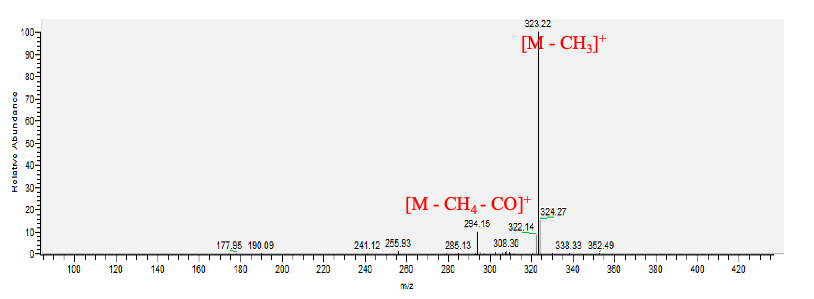
^

**Compound 8:** berberine

MS^1^

##
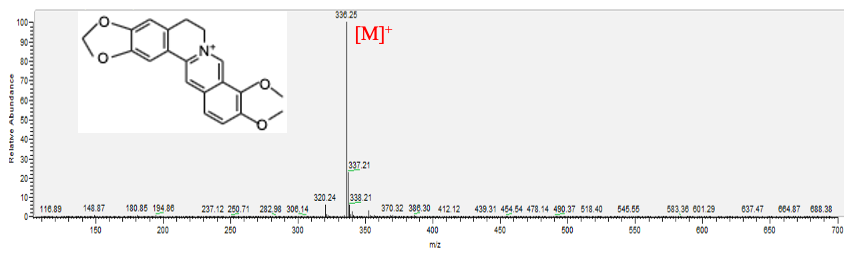


MS^2^

**
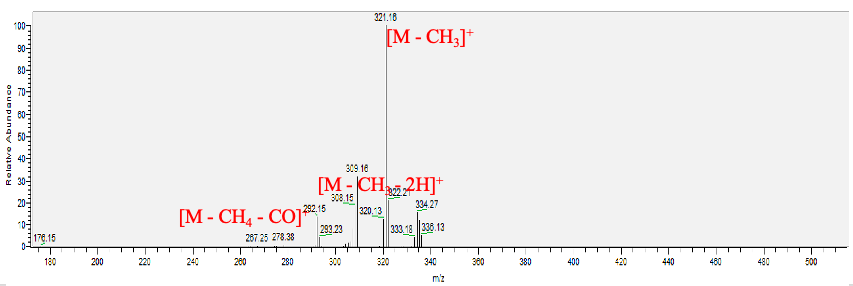
**

**Compound 9:** dehydrocorybulbine

MS^1^


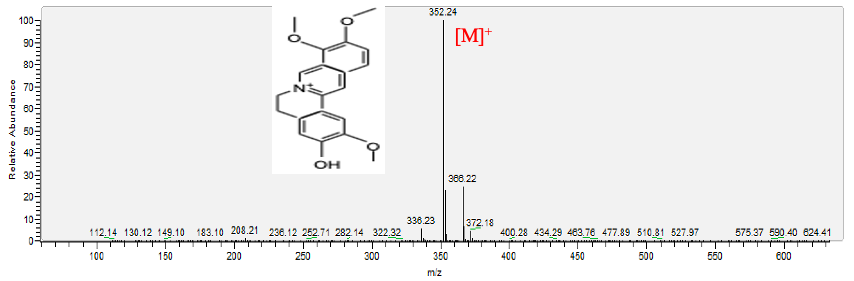


MS^2^

**^
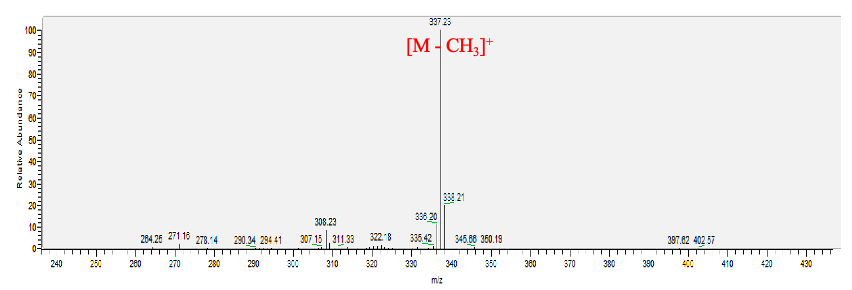
^**

**Compound 10:** palmatine

MS^1^

**
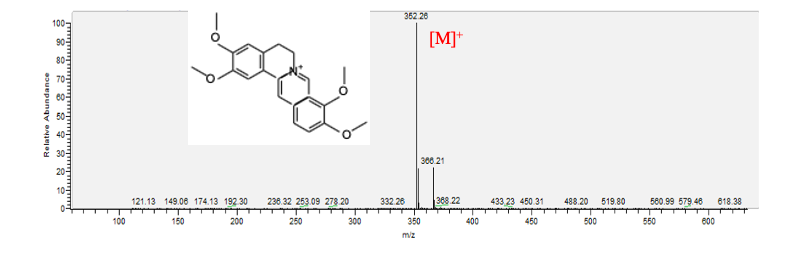
**

MS^2^

###
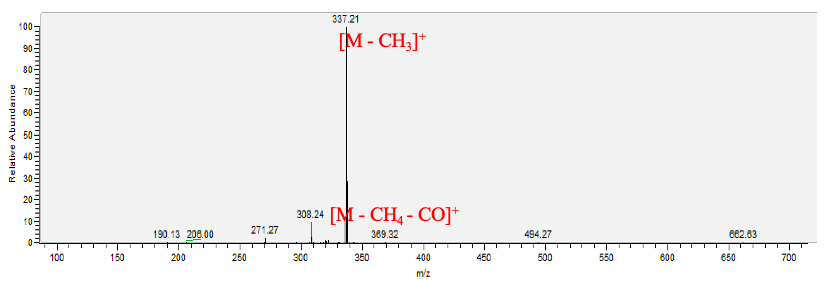


**Compound 11:** dehydrocorydaline

MS^1
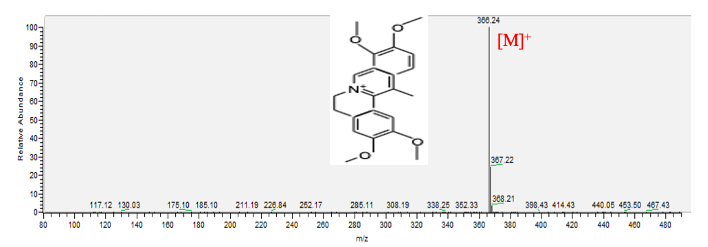
^

MS^2^

**
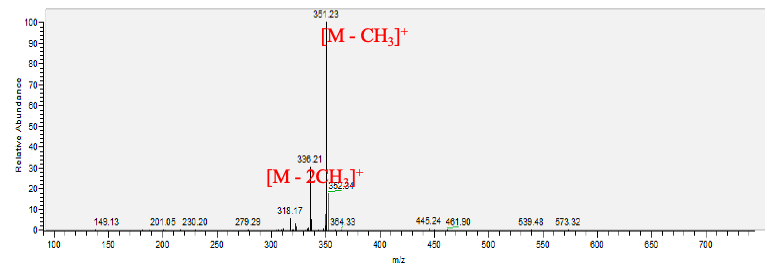
**

**Compound 12:** oxoglaucine

MS^1^

**
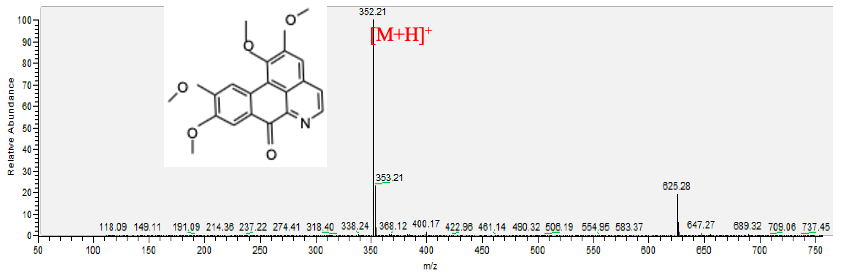
**

MS^2^

**
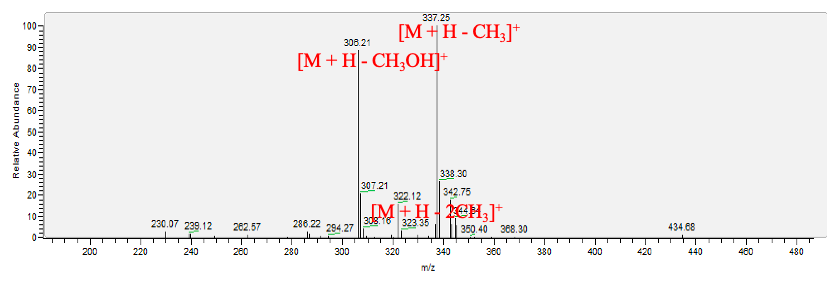
**

**Reference**

Sun, M., Liu, J., Lin, C., Miao, L., and Lin, L. (2014) Alkaloid profiling of the traditional Chinese medicine Rhizoma corydalis using high performance liquid chromatography-tandem quadrupole time-of-flight mass spectrometry. *Acta Pharm. Sin. B.* 4, 208-216. doi:10.1016/j.apsb.2014.04.003

Wang, M., Liu, Y., Fu, S., Zhang, Q., Wang, Q., and Gao, X. (2017). Applying target data screening followed by characteristic fragment filtering for the comprehensive screening and identification of alkaloids in *Corydalis yanhusuo* W. T. Wang by UPLC-Q-TOF/MS^E^. *RSC Adv*. 7, 53545-54551. doi:10.1039/c7ra0
